# Transactions of the Association of Army and Navy Surgeons

**Published:** 1864-04

**Authors:** 


### TRANSACTIONS OF ASSOCIATION OF ARMY AND NAVY SURGEONS.

Association of Army and Navy Surgeons, Feb. 13th, 1864.

Surgeon-General S. P. Moore, President, in the chair.

The minutes of the last meeting were read and approved.

Letters were read from members of the Association in reply to the questions on tetanus: From Surgeon J. G. Dudley, giving the history of a case of tetanus of the mildest kind, in which the spasms were almost null, confined to the muscles of the jaws and neck, with only one severe spasmodic act, which lasted but a short while, then wore off, allowing the free administration of fluids and nourishment—yet the patient gradually sank on the tenth day after the seizure. This case occurred in the fifth week from the receipt of an injury in the great toe. This was the only case in his division of Winder Hospital out of 1,400 wounded under treatment since its organization. From Surgeon Read, seven cases of tetanus, with five deaths and two recoveries. An analysis of twenty-six cases from the statistical bureau, from Surgeon H. Baer.

The President called for the report of the committee on gun-shot wounds of the chest.

Surgeon Thom, chairman of the committee, read an interesting report, in which was considered: The general treatment of injuries of the lungs from missiles, penetrating and cutting weapons; the time and manner of death under such circumstances; the pathological condition, functional embarrassment, or usefulness remaining after these accidents; the mode of production and treatment of emphysema; and the provisions made by nature for accommodating foreign bodies retained within these organs, with the amount of disturbance which ensues. He regretted that few replies had been received to the interrogatories which the preparation of this report had suggested, and that he could furnish only seventy-four cases of gun-shot wounds of the lungs, in which twenty recovered, from which limited number it appeared the mortality was little over twenty-five per cent., or one-quarter. As far as could be ascertained, bleeding had been resorted to in but one case, and that recovered. He mentioned a case of recovery stated by Surgeon W. G. Semple, where a gig shaft passed entirely through the right lung of a man, reminding us of a case recorded by Mr. Madin, of Stratford, England, in which the thorax was entirely transfixed by a gig-shaft that entered under the left arm and passed out beneath the right, perforating both lungs. The reporter had seen several cases in which balls were retained within the lungs with very trifling inconvenience, and cited in particular the case of J. W. Burke, company "C," 6th Virginia regiment, wounded at Malvern Hill, July 1st, 1862. The musket ball entered half an inch above left nipple. The recovery was rapid. Transferred to ordnance works in this city; his general health remains excellent, though he is unable to bear active exercise without difficulty of breathing; cannot lie on left side, and suffers occasionally from pain in left side of chest and under scapula of the same side. This report was concluded by the history of a most remarkable case of gun-shot wound of lung and extraordinary recovery, communicated through letters received from Surgeons W. Selden and W. J. Moore. Mr. R. D. Q., 22 years old, of scrofulous temperament, in January, 1840, was leaning on his gun, the muzzle in contact with his left side, when it exploded, tearing a hole in the chest of three or four inches in diameter, carrying with the load of shot fragments of the third, fourth and fifth ribs, and the whole of a very large, heavy English gold patent-lever watch, except the ring to which the chain was attached, which, singular to say, was found in the lining of his waistcoat, on the right side. Dr. Selden found the patient apparently about to expire, and, from the impending suffocation upon the ingress of air within so large an opening, he could make no exploration of the wound. Closing the wound with a large compress and bandage, opium and stimulants were freely administered. Re-action took place, and in a fortnight sufficient adhesions were established to permit exposure of the cavity of the wound and to recognize and remove the metal face of the watch from some six inches at the bottom of the wound. For several weeks fragments of the watch continued to present themselves and were extracted, some from upon the diaphragm, others below the clavicle. The lung collapsing, was not torn to pieces, though wounded in several points. Both the heart, covered by the pericardium, and the aorta, were exposed to view and to touch. Suppuration was enormous; hemorrhages frequent. The collapsed lung became bound down by adhesions. The whole side of the thorax sank. Sustained by every article of nutritious food calculated to supply an inordinate appetite, the patient's recovery was slow, until the wound, progressively reduced, could only admit a female catheter. The supervention of the *tentement metallique* during the progress of the case offered the enviable opportunity of viewing the cause of its production. Drs. Andrews and Higgins, (whose patient Mr. D. was,) were perfectly assured that the bursting of the bubble on the surface of the pus was the rationale of the sound. Fragments of watch and bone together, with shot and other extraneous matters, continued for some time to be ejected by expectoration with sputa. Mr. D. possesses, now, every part of the watch except the hands, a considerable portion of the *small works* having been expectorated. The openings into lung were of sufficient size to allow a current of air to escape, and if directed against the flame of a candle to extinguish it. Mr. D.'s health continues feeble, but is as robust as it had been during the past five years.

The President called for any voluntary communications from members present, saying that if there were none, the topic for discussion was still before the society.

Surgeon Michel said he hoped the Society would indulge him while he reverted to a subject which, he was more convinced than ever, required consideration from the doubt that had been expressed, whether any real difference existed between tetanic spasms in a part and the disease termed tetanus. He would not allude again to the important bearing of this entire subject upon the record of cures, or even the determination of the number of cases of tetanus; but begged to examine the general proposition, *that every partial muscular contraction must, of necessity, depend upon reflex action.* Certain physiological principles were involved in this proposition, and their apprehension furnished, perhaps, grounds for the distinction he recognized between the symptoms of a hopeless disease, and those spasms or contractions of a benign character in muscles of a limb from traumatic or idiopathic causes. Muscular contractility was a property inherent to the tissue itself. Muscular fibre was potentially endowed with the power to contract independent of nerve force. Muscles in an amputated part were seen to contract, and this motility could not be referred to irritation of the nervous filaments within the muscular structures; for, after galvanism and electricity applied to these nerves had so exhausted the nervous force as no longer to elicit any response, the slightest irritation, mechanical or chemical, applied to the muscles themselves, at once developed contractions. Physiology, moreover, showed that muscular irritability was of longer duration than nervous irritability. Cadaveric rigidity, supervening sometime after death, was, he believed, another exhibition of muscular action without nervous agency, since it occurred even in paralysis, provided no atrophy or other nutritive change took place in the muscles. But the most remarkable example was the rhythmical movements of the heart, when this organ is removed from the body of a living animal. Its contractions and dilatations continue for a length of time without the possibility of invoking reflex spinal influence, as explanatory of their production. He would not raise the question of the existence of *ganglia*—those magazines of nervous force in the body of the heart—for the same pulsations are seen in the rudimentary heart of the embryo, when the heart is as yet but a blastema of cells, and before the development of any nervous apparatus. The nerves were the ordinary channels through which muscles were brought into play, but they constituted *only one* of the channels. The blood, itself, maintaining the vitality of the muscular tissue, and its inherent irritability, becomes at once *another* channel for the induction of muscular contraction, if freighted with any *noxious* principle adequate to its production. In these spasms, if the main blood-vessel be ligatured, the contracting ceases in the limb thus operated on. If, therefore, excitation could be conveyed by any other route than through the nerves of the part, muscular irritability once aroused would declare itself in local contractions of the organs thus stimulated. It was not impossible to conceive that such changes should occur in the wounded limb, amidst the wreck of all the tissues, as would introduce some morbific elements into the blood of the part calculated to awaken a certain degree of irritation in the muscular structures, whose partial contraction would be expressive of a local condition, not necessarily indicative of a nervous lesion sufficient to engender spinal irritation—not, therefore, a manifestation of reflex action. Dr. M. said he made these suggestions with the view to direct attention to the want of normal relation that might exist between the blood and the tissues through which this blood was circulating, and the results which possibly might follow; and to guard against calling nervous lesions always into requisition to account for the phenomenon of spasm. Were these spasms not sometimes seen to occur where no lesion of any kind existed? He would instance cramp in the calf of the leg, and hiccough. These were of idiopathic origin, indicating some perturbed condition of the individual muscles themselves. Every one had experienced a twitching or quivering of the orbicular muscle of the eye, at times most annoying, and from no appreciable cause; confined frequently to one portion only of that muscle, in either the upper or lower eye-lid. The cramps in leg during the partrurient act were readily accounted for by pressure of the child's head upon the plexus of nerves, but in the other instances mentioned, we seemed conscious that the muscles themselves were irritated and discharging their motivity.

Surgeon Campbell said that he would remark, in relation to Dr. Michel's suggestions, that while he had little doubt that the tendency to spasmodic action in a wounded limb is, in a great measure, favored by that increased muscular irritability consequent upon the hyperæmia and general exalted action of the part, and while he would be glad to know that careful investigations were in process, to determine the exact relations which this condition of the muscular fibre sustained to the phenomena observed, not only in traumatic spasms, but in tetanus itself, he would be unwilling, without the most thorough examination and discussion of the subject, to accept such a discrimination as the basis of classification for tetanus and traumatic spasms. It seemed to him more reasonable to conclude that, in the evokement of *all* spasmodic actions, whether tonic or clonic, the *same apparatus* was brought into reqisition, viz: an excitor nerve, a spinal centre, and a motor nerve. The condition of the muscles acted on, through these agencies, might modify in any given case, to a certain degree, the *character* of the phenomena, but, in his opinion, the phenomena presented by convulsive actions of every kind are such, that we could not legitimately attribute them *solely* to enhanced muscular irritability. The subject of muscular irritability was one of great interest in its relations to gun-shot wounds, and he hoped his remarks would not be discouraging the discussion of it in connection with the subject under debate.

Surgeon Lewis remarked that he had recently seen a patient who, after premonitory symptoms of general uneasiness and pain from gun-shot wound, was seized with spasmodic movements of the muscles of neck and side of face, extending down the arm. The muscles contracted intermittently in this way and then relaxed. The patient died. Was this tetanus or not? [The President inquired whether the patient had exhibited any trismus?] There was no permanent rigidity of the jaws, though their muscles were at times contracted.

Surgeon Reid observed that, as to trismus, he had seen the disease begin in the wounded limb and progress to trismus, and the patient relieved by free incisions around the wound. Again, in a certain case of gun-shot wound of the hip, complete tetanus occurring, digital exploration invariably brought convulsions of the leg *only*. He believed in the importance of destroying the connection of the injured nerve with the centre.

Surgeon Bolton thought it important not to allow similarity of symptoms to impose the belief that the case was one of true tetanus. He had treated a patient, whose foot was injured by a rusty nail, who had symptoms strikingly like tetanus with trismus, which, as the disease developed itself, proved to be a severe cold, accompanied with swelling of the cervical glands and coryxa, with ulcers in the throat. At certain seasons of the year, we meet with cases of cholera of the severest character, assuming all the appearances of Asiatic cholera, yet no one supposes that the two diseases are identical.

Surgeon Hancock stated that, after the battle of Drainsville, no less than six cases of tetanus occurred among only seventy wounded. He was satisfied that atmospheric changes exerted on this occasion some striking influence upon the development of the disease, for the wounded had lain exposed upon the field under a very severe change within the twenty-four hours succeeding this engagement. That, perhaps, Dr.

WILLIAMS remembered the circumstances, as medical director at the time.

Surgeon WILLIAMS replied, that these cases, which he remembered well, were noted by him at the time, and sent on to the General Hospital; that he had not learned the result.

The hour for adjournment having arrived, the President dissolved the meeting.

